# Gastric infiltration of hepatic sarcomatoid carcinoma: A case report and literature review

**DOI:** 10.3389/fsurg.2022.1031284

**Published:** 2023-01-06

**Authors:** Shuoshuo Ma, Dengyong Zhang, Guanru Zhao, Sheng Ding, Qiong Wu, Xueli Zhang, Zheng Lu

**Affiliations:** ^1^ Department of General Surgery, The First Affiliated Hospital of Bengbu Medical College, Bengbu, China; ^2^ Department of Pathology, Hospital of Bengbu Medical College, Bengbu, China; ^3^ Department of Radiology, Hospital of Bengbu Medical College, Bengbu, China

**Keywords:** hepatic sarcomatoid carcinoma, gastric stromal tumor, case report, infiltration, review

## Abstract

**Background:**

Hepatic sarcomatoid carcinoma (HSC) is an extremely rare malignant tumor typically observed in clinical settings. HSC occurrence is predominantly noted in the right lobe and rarely in the left lobe of the liver. This report presents a case of sarcomatoid carcinoma that occurred in the left outer lobe of the liver, which was accompanied by gastrointestinal stromal tumors (GSTs) in the greater curvature of the stomach. In addition, the patient showed late-stage recurrence of HSC in gastric tissues.

**Case presentation:**

A 63-year-old man was concomitantly diagnosed with HSC and GST. The main clinical manifestation was fever. Abdominal computer tomography (CT) and ultrasound-guided percutaneous liver biopsy at the local hospital revealed the presence of malignant hepatic tumors. The patient approached our hospital for further treatment. The subsequent electronic gastroscopy showed multiple submucosal tumors (SMT) in the stomach. Owing to the absence of multiple metastases in other regions of the body, we performed left hepatic lobe resection with gastric partial resection. The postoperative pathological analysis confirmed the presence of HSC and GST. The patient reported feeling well 1 month after the surgery, and no obvious space-occupying lesions in other areas were noted *via* imaging examinations. However, 3 months later, the patient presented with pain in the upper left abdomen, and examination revealed cancer recurrence in the stomach. The surgery was repeated, and the patient recovered favorably after the procedure. Unfortunately, the patient died owing to multiple metastatic diseases 4 months after the second surgical procedure.

**Conclusion:**

HSC shows no characteristic clinical manifestations and is highly malignant. Surgical intervention is the first treatment of choice for patients with HSC. In cases of sarcomatoid cancer occurring in the left lobe of the liver, it is imperative to exercise strict vigilance against the tumor's invasion of the stomach tissue. This is particularly important when the tumor breaks through the capsule of the liver.

## Introduction

Hepatic sarcomatoid carcinoma (HSC) is a particularly malignant tumor with unknown pathogenesis, and it accounts for 2% of surgically removed cases of hepatocellular carcinoma (HCC) and 3.9%–9.4% of autopsies (1, 2). HSC may be a tumor of monoclonal origin; however, its histological origin remains controversial (3). Compared with HCC and intrahepatic cholangiocarcinoma (ICC), HSC has a higher histological grade, recurrence rate, and metastasis rate; therefore, its prognosis is extremely poor. Currently, surgical treatment is the first choice of treatment, and adjuvant treatments include radiotherapy, chemotherapy, and interventional therapy among other comprehensive approaches.

In the current report, we present the case of a 63-year-old man who showed fever as the main clinical manifestation and had a postoperative pathological diagnosis of HSC and gastrointestinal stromal tumors (GST). We performed left hepatic lobectomy combined with partial gastrectomy on the patient, and the patient recovered favorably after the operation. However, 3 months later, the patient presented with gastric recurrence and received surgical intervention for the second time. In this paper, we share the clinical characteristics of and the diagnosis and treatment process for this patient with the aim to assist colleagues in their associated work.

## Case presentation

### Clinical history and laboratory findings

At the time of hospital admission, the patient was a 63-year-old male and showed fever as the main clinical manifestation. His body temperature fluctuated in the range of 37.5°C–38°C. The patient did not have jaundice, the abdomen was flat and soft, and showed no tenderness or rebound pain. The liver and spleen were not palpable under the ribs, and no mobile dullness was identified. The patient did not have a history of chronic diseases such as hypertension and diabetes, a history of infectious diseases including hepatitis B and tuberculosis, or a family history of tumors.

Blood routine tests showed increased levels of white blood cells (11.14 × 10^9^/L). The blood biochemical examination revealed that alanine aminotransferase (37 U/L) and aspartate transferase (18 U/L) were within the normal range. We noted a decrease in the total protein content (62.8 g/L) and the white ball ratio (1.0); however, the C-reactive protein (215.70 mg/L) levels had increased abnormally. Immune screening detected no hepatitis B virus (HBV) or hepatitis C virus (HCV) infection. Through the tumor marker screening, we determined that carcinoembryonic antigen (CEA) (1.24 ng/ml), alpha-fetoprotein (AFP) (4.47 ng/ml), prostate-specific antigen (2.98 ng/ml), carbohydrate antigen 19–9 (CA19–9) (6.32 IU/ml), and carbohydrate antigen 15–3 (CA15–3) (18.10 IU/ml) were within the normal range.

### Imaging examinations

The patient underwent a physical examination at the local hospital. Ultrasound examination of the liver, gallbladder, pancreas, and spleen showed the presence of space-occupying lesions in the left lobe of the liver. Computed tomography (CT) of the upper abdomen indicated a liver abscess and multiple lymphadenopathies in the abdominal cavity. However, a subsequent (1 week later) ultrasound-guided percutaneous liver biopsy revealed a malignant tumor in the left lobe of the liver.

**Figure 1:**
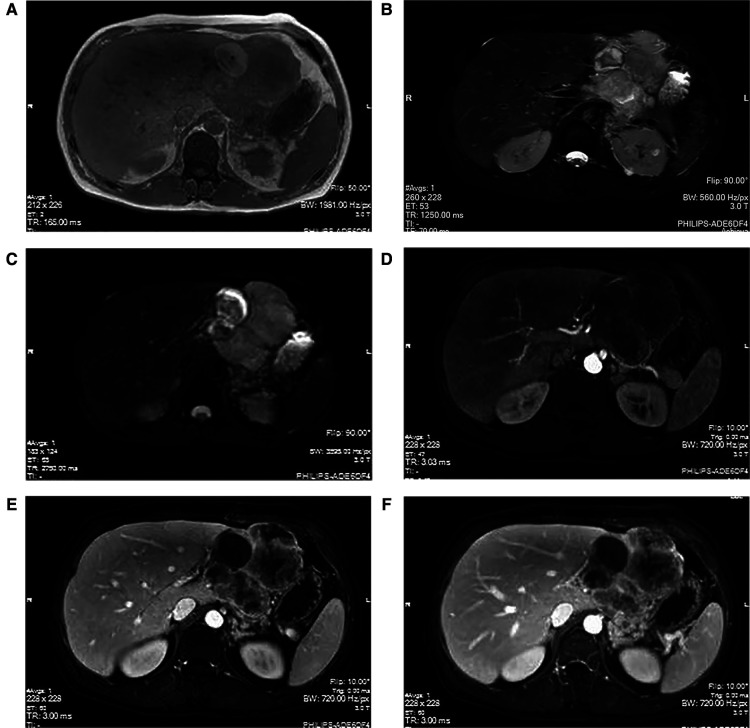
MRI manifestations of hepatic sarcomatoid carcinoma. The magnetic resonance imaging (plain scan + enhanced + functional imaging) shows abnormal signals of irregular mass in the liver, with a size of approximately 9.5 × 8.3 cm. The T1WI (**A**) shows a weak signal; however, the T2WI (**B**) and the DWI (**C**) show strong signals. The arterial phase (**D**) indicates slightly uneven enhancement at the edge of the lesion, with continuous uneven enhancement in the portal phase (**E**) and delayed phase (**F**).

To receive further treatment for liver tumors, the patient was admitted to the Department of Hepatobiliary Surgery at our hospital. The Color Doppler ultrasound (hepatobiliary, pancreatic, spleen, and portal vein) showed that the left outer lobe of the liver had a mixed echo of 103 × 112 × 110 cm. The tumor was mainly hypoechoic with an irregular shape and clear boundaries. The internal echo was uneven, the color revealed the presence of dots of blood flow signals in and around the tumor, and no abnormality in the portal vein was noted. Irregular masses of long T1 (Figure 1A) and long T2 (Figure 1B) abnormal signals in the space between the liver and the stomach were detected through magnetic resonance imaging (MRI) of the tumor (plain scan + enhanced + functional imaging). In the images, the tumor size was approximately 9.5 × 8.3 cm, the circular short T1 and long T2 signals can be clearly visualized, and the tumor has a well-defined boundary. However, the lesion and the left lobe of the liver appear to be unclearly decomposed. Diffusion-weighted imaging (DWI) (Figure 1C) shows a strong signal, and the enhancement (Figures 1D–F) indicates progressive heterogeneous enhancement. Irregular thickening of the stomach wall was noted on the side of the corresponding lesser curvature of the fundus. The gallbladder was not enlarged, and its wall did not appear to be thickened. No dilation was observed in the intrahepatic and extrahepatic bile ducts and we noted no obvious filling defect in the main portal vein and its branches. We did not identify any abnormalities in the pancreas and spleen morphology or any obvious signal indicating irregularity. In the imaging range, multiple small lymph nodes could be identified in the retroperitoneum. Chest x-ray and CT images showed no abnormalities in the lungs, the heart, or the diaphragm. We used an electronic gastroscope and determined the presence of a submucosal bulge with a diameter of approximately 0.8 cm in the fornix and the greater curvature of the stomach. The surface of the protuberance was smooth, tough to touch with the biopsy forceps, and appeared to be inactive. This observation suggested that there were multiple SMTs within the protuberance. In the lesser curvature of the stomach near the corner, we noted that there was a sheet-like shallow erosion with smooth mucosa flushing in color; however, no ulcer or bleeding was observed. Pathologic examination of the biopsy revealed chronic superficial gastritis of the mucosa, which was severe, active, and locally accompanied by ulcers and *Helicobacter pylori* (HP) infection.

### Intraoperative situation

The patient was febrile and showed no characteristic clinical presentation. Results of laboratory tests, including tumor markers, were unremarkable. A combination of imaging findings and liver puncture results of the patient in the local hospital assisted us in determining that the patient had a malignant tumor in the left lobe of the liver, accompanied by greater curvature GST, without obvious metastatic lesions. Owing to the fact that only a small amount of pathological tissue was acquired through the liver puncture, the nature of the tumor required further elucidation *via* postoperative pathologic analysis. HCC, ICC, HSC, and other properties are not excluded. We performed left hepatic lobectomy combined with partial gastrectomy (wedge resection) with the patient's and his family's consent. We intraoperatively identified a mass of ∼10 cm in diameter in the left lobe of the liver, with off-white color and hard texture. The tumor had appeared to have broken through the liver capsule, the visceral surface densely adhered to the lesser curvature of the stomach, and enlarged lymph nodes were palpable in the suspensory ligament of the hepatoduodenum. Intraoperative biopsies were performed and all three enlarged lymph nodes tested negative upon pathologic examination. Two superficial tumors were observed on the greater curvature of the stomach wall. The right liver was normal in size and soft to the touch. The size of the gallbladder was approximately 7 × 4 × 3 cm. No tumor metastatic nodules were detected in the omentum, diaphragm, jejunum, or pelvis. Intraoperative examination revealed that the liver tumor had invaded the left gastric artery, which was removed from the root. The operation was relatively successful, lasted for approximately 6 h, and the bleeding volume was approximately 200 ml.

### Pathology

**Figure 2:**
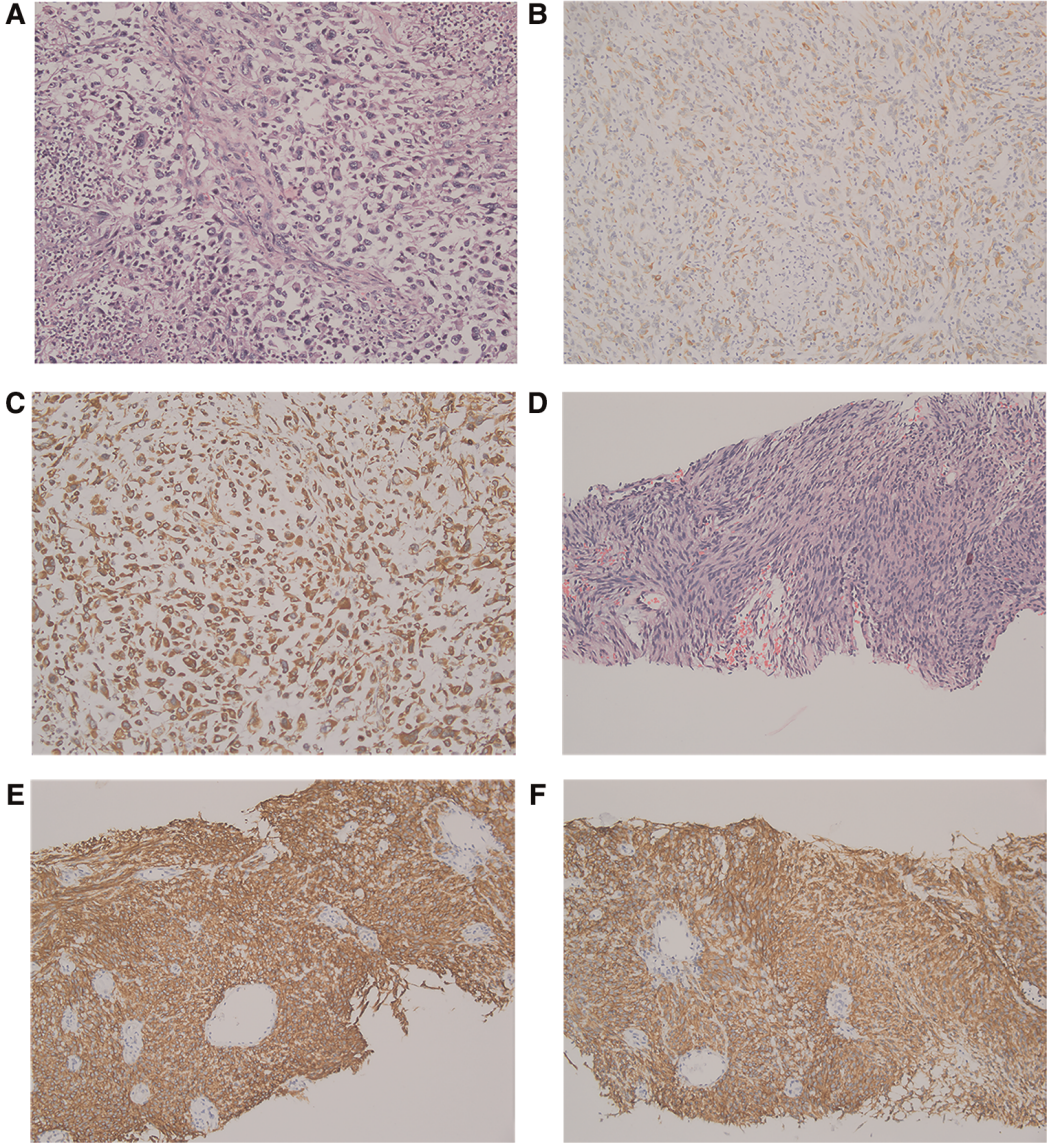
Pathology and immunohistochemistry of hepatic sarcomatoid carcinoma and gastrointestinal stromal tumors. Hematoxylin and eosin (H & E) staining of hepatic sarcomatoid carcinoma (**A**); the results of immunohistochemical staining of hepatic sarcomatoid carcinoma show CK positive ×200 (**B**) and AE1/AE3(+) positive ×200 (**C**); H&E staining of gastrointestinal stromal tumor (**D**); the results of immunohistochemical staining of the gastrointestinal stromal tumor shows CD117 positive ×200 (**E**) and DOG-1 positive ×200 (**F**).

The postoperative specimens were subjected to pathological examination and immunohistochemistry (IHC). The liver tumor tissue specimen was diagnosed as a high-grade pleomorphic malignant tumor of the left lateral lobe of the liver with necrosis. The immunological markers were as follows: CK (+) (Figure 2B), Vim (−), CD117 (−), Dog-1 (−), CD34 (−), SMA (−), Ki-67 (+, ∼60%), GPC-3 (−), and Hepar-1 (−). Additionally, the tissue specimen was sent to another hospital for consultation, and the IHC results showed AE1/AE3 (+) (Figure 2C), P16 (−), CDK4 (+), MDM2 (−), Desmin (−), Myogenin (−), and MyoD1 (−). Molecular detection: MDM2 gene status (−), no amplification. According to the outcomes of IHC analysis, a sarcomatoid carcinoma (SC) with a length of 10 cm, and a negative cut-off edge of the liver section was considered. The gastric tissue specimen was diagnosed as representing spindle cell proliferative disease, combined with immunological markers in line with low-risk GST, featuring 2 pieces with a length of 1.0–1.5 cm. The relevant immunohistochemical markers were as follows: Vim (+), CD117 (+) (Figure 2E), CD34 (+), Dog-1 (+) (Figure 2F), Ki-67 (+, ∼5%), SMA (−), S-100 (−).

### Outcome and follow-up

The patient recovered favorably and was discharged from our hospital on postoperative day 8. No adjuvant therapy was administered or prescribed. The patient was followed up in the outpatient clinic 1 month after discharge, and he presented no obvious symptoms or discomfort. The blood routine test and biochemical profile showed no abnormalities, and AFP (10.5 ng/ml) had no significant increase compared with the first measurement (4.47 ng/ml). The hepatobiliary, pancreatic and splenic ultrasound showed no obvious space-occupying lesions.

**Figure 3:**
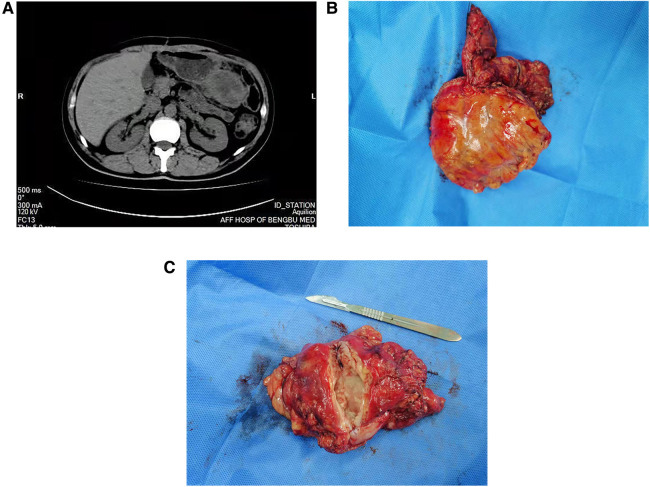
Related imaging data and clinical specimens of gastric recurrence of hepatic sarcomatoid carcinoma. A regular CT scan of the abdomen showed the presence of an irregularly shaped and uneven-density occupancy in the area between the greater curvature of the stomach and the omental sac (**A**), the size was approximately 53 × 78 × 100 mm; the specimen is off-white (**B**) and shows internal necrosis (**C**).

However, 3 months later, the patient presented with persistent pain in the upper left abdomen, which worsened paroxysmally. Imaging examinations revealed the presence of an irregular and substantial mass in the area between the greater curvature of the stomach and the omental sac (Figure 3A), and the enhanced CT scan showed uneven enhancement. The patient underwent gastric tumor resection with partial resection of the transverse colon. The tumor was 53 × 78 × 100 mm in size, gray in color, and showed internal necrosis (Figures 3B,C). Results of postoperative pathology and IHC tests confirmed that the tumor in the stomach had metastasized from the HSC. The patient recovered well after the operation. Four months after the second operation, the patient died from multiple metastases to other sites.

## Discussion and conclusion

Cases of SC are extremely rare in clinical practice. SC may occur in multiple organs throughout the body and even occur concurrently. The lung and the bladder are the most common sites of occurrence (4, 5), whereas cases presenting with SCs in the liver are rare. HSC is a rare malignant tumor that typically occurs in the right lobe but rarely in the left lobe of the liver. HSC accounts for 1.8% of all surgically resected HCCs and 3.9%–9.4% of autopsy cases (1, 5). The age of onset of HSC patients is approximately 60 years, and the disease is more common in men, with its incidence 3–4 times that of women (6, 7). Herein, we have reported the case of a 63-year-old male patient, which is consistent with the characteristics of previous reports. HSC etiologies and pathogeneses require further elucidation; however, two main schools of thought exist in the medical field. One states that HSC is the sarcomatoid transformation of HCC, whereas the other states that it is a combination of liver cancer and sarcoma. However, among the two, the former is more widely accepted by scholars (8). Previous studies have reported that sarcoma transformation may be related to certain preoperative anti-liver cancer treatments, including transcatheter hepatic artery chemoembolization (TACE), radiofrequency ablation (RFA), and percutaneous ethanol injection therapy (9–11). Conversely, in our case, the patient had no history of treatment. Under normal circumstances, the volume of HSC is larger than that of HCC, which helps to distinguish between the two. In a study of 28 HSCs, the average HSC size was 6.1 ± 2.8 cm, and 18 cases (64.3%) had tumors larger than 5 cm (12). Another study involving 17 cases of HSC pointed out that its long diameter was 6.12 ± 3.18 cm, whereas the HCC's long diameter was 4.21 ± 2.38 cm (13). In our case, the HSC had a long diameter of approximately 10 cm, which was consistent with the previous reports. For larger HSCs, the probability of false capsules and bleeding may increase. In the study including 28 patients, 9 (32.1%) had pseudocapsule formation, most of which were incomplete (12). HSC is highly aggressive and is associated with a particularly poor prognosis. In the abovementioned study, the researchers noted that up to 47.1% of HSC patients had intrahepatic metastasis, which was considerably greater than the 12% of HCC patients; however, this value was not significantly different from that of ICC (13). A total of 93% of HSC patients reportedly presented with extrahepatic metastases during treatment, with the most common metastatic organs being the lung, peritoneum, pleura, pancreas, adrenal gland, intestine, and spleen. A study with 28 patients reported that the number of patients with local invasion, vascular invasion, bile duct invasion, and lymph node metastasis was 6 (21.4%), 8 (28.6%), 3 (10.7%), and 7 (25%), respectively (12). Similar characteristics were observed in other study (14). In the present case, no other organ metastases were observed at the first visit; however, the tumor invaded the left gastric artery and gastric recurrence appeared 3 months after surgical intervention. A study involving univariate analysis (12) highlighted that the TNM stage, pseudocapsule formation, local invasion, vascular invasion, Child–Pugh classification, and radical resection are the prognostic factors of OS in HSC patients. A study involving 79 cases of HSC reported that the 1-year and 3-year overall survival rates were 63.3% and 35.4%, respectively (15). In addition to these poor survival rates, HSC is prone to recurrence. Notably, during the follow-up period, 25 of 28 (89.3%) patients who underwent radical surgical resection showed tumor recurrence, and the cumulative recurrence rate at 6, 12, and 24 months was 53.6%, 78.6%, and 85.7%, respectively (12).

Symptomatic HSC cases account for 68%–75% of cases. Abdominal pain is the most common symptom (7, 8, 12), followed by fatigue, fever, and jaundice. The typical systemic symptoms caused by liver decompensation are jaundice, fever, abdominal distension, and weight loss. In addition, patients with HSC may present with gastrointestinal symptoms, including persistent pain in the upper right quadrant, nausea, vomiting, weight loss, and palpable mass in the xiphoid (16). Our patient had fever as the main clinical presentation, which is common in other diseases. In addition, a small percentage of patients are asymptomatic (17.6%). To a certain degree, this observation is the opposite of liver cancer, which tends to exhibit no symptoms for most patients (70.0%) (13). Furthermore, 14 of 19 HSCs (73.7%) reportedly had a history of liver cirrhosis. A number of studies have also pointed out the potential relationship between chronic liver disease and HSC (17–19). However, in our case, liver function indicators (ALT and AST, among others) and tumor indicators (AFP and CEA, among others) were in the normal range. This is different from the features of liver cancer that we have observed previously, and it caught our attention.

On an enhanced MRI image, HSC is typically manifested as peripheral enhancement, central necrosis, and different enhancements with and without a tumor envelope in the solid part (20). The varied tissue composition of HSC determines its mode of enhancement; therefore, this indicates that the imaging results of HSC are complex and diverse. In one example, more than half of HSC cases showed progressive enhancement in the Gd-DTPA-enhanced MRI scan, and approximately one-third of cases showed continuous high enhancement (20). Another study involving 10 cases of HSC reported that the enhancement mode of MRI was mainly “progressive” (21). The continuous enhancement mode of HSC is one of the crucial points to successfully identify the presence of HCC. This is attributable to the fact that the latter is primarily manifested as the “fast in and out” of the contrast agent. In our case, the T1WI showed a strong signal, the enhancement showed progressive unevenness and the lack of a “target sign.” Although this is similar to previous reports, further differentiation from ICC should be noted. In addition, intrahepatic metastasis (17, 18), lymphadenopathy (13), and adjacent biliary tract dilation (19, 22) may assist in distinguishing between HSC from HCC as these characteristics are predominantly observed in HSC.

An accurate HSC diagnosis relies on a large number of pathological signs and IHC observations. HSC is composed of spindle cells and epithelial components that are identifiable in ultrastructure, IHC, and morphology (23). The IHC staining results of HSC were positive for the sarcoma-like component marker Vimentin (Vim), the epithelial component markers keratin (CK) and epithelial membrane antigen (EMA) (24, 25) as well as the anion exchanger (AE) 1/AE3, 34*β*E12, CAM 5.2, c-Kit, S-100 protein, HHF-35, kinesin-like protein-1, CD34, and HAM-56, which is helpful for the diagnosis of HSC (26). In the present case, the epithelial component markers keratin (CK) was negative; however, AE1/AE3 was positive. This is worth our attention. In cases when the microscopic features tend to indicate SC, but the IHC indicators appear to be inconsistent, adding other IHC indicators should be prioritized.

Reportedly, surgical resection has been an effective method for the treatment of HSC. Radical surgery can significantly prolong the overall survival of patients with HSC, and the associated survival period is significantly higher than that of palliative resection (12, 14). In the present case, we performed radical resection of the tumor. Unfortunately, the possibility of invasion of the gastric tissue by the tumor was overlooked, this was despite the fact that endoscopic biopsy suggested a negative result. This may have been the main etiology responsible for the subsequent gastric recurrence in the patient. For unresectable tumors, systemic chemotherapy and local radiotherapy can be selected (27). For multiple metastases, combined treatments, such as radiotherapy, chemotherapy, and hyperthermia, can alleviate symptoms, but they cannot change the overall survival (OS) (16). Owing to the poor differentiation of carcinogenesis and the aggressiveness of HSC, recurrence may be likely even in the early stages of the disease. Vascular invasion and local invasion are independent risk factors for short disease-free survival (DFS) (12). In this case, invasion of the left gastric artery and infiltration of the gastric wall may be important reasons for the recurrence and short survival period of our patient. Although previous studies found that HSC is mainly manifested as vascular clumps, adjuvant TACE is considered a risk factor related to poor prognosis (12). However, among patients with intrahepatic recurrence after hepatectomy, the median survival time of those who received TACE treatment was 14.6 months, which was significantly longer than the 8.1-month survival time of patients who received supportive treatment alone (12). These findings provide evidence that TACE may be beneficial for localized intrahepatic recurrence and may improve the survival rate of these patients.

HSC is highly malignant; thus, timely detection, accurate diagnosis, and personalized treatment are of paramount importance to positively impact disease prognosis. In cases presenting with SCs in the left lobe of the liver, clinicians should be vigilant regarding the possibility of gastric infiltration; this is a plausible outcome if the carcinoma breaks through the liver capsule. If available, gastric endoscopic ultrasonography should be used to evaluate the involvement of the gastric wall. In this report, we have shared our patient's clinical characteristics and the corresponding diagnosis and treatment process to provide a reliable reference for healthcare professionals in this field.

## Data Availability

The original contributions presented in the study are included in the article/Supplementary Material, further inquiries can be directed to the corresponding author/s.

## References

[1] Kakizoe S Kojiro M Nakashima T. Hepatocellular carcinoma with sarcomatous change. Clinicopathologic and immunohistochemical studies of 14 autopsy cases. Cancer. (1987) 59(2):310–6. 10.1002/1097-0142 (19870115)59:2<310::aid-cncr2820590224 > 3.0.co;2-s 2433017

[2] Giunchi F Vasuri F Baldin P Rosini F Corti B D'Errico-Grigioni A. Primary liver sarcomatous carcinoma: report of two cases and review of the literature. Pathol Res Pract. (2013) 209(4):249–54. 10.1016/j.prp.2013.01.005 23484778

[3] Thompson L Chang B Barsky SH. Monoclonal origins of malignant mixed tumors (carcinosarcomas): evidence for a divergent histogenesis. Am J Surg Pathol. (1996) 20(3):277–85. 10.1097/00000478-199603000-00003 8772780

[4] Kadouri Y Ouskri S Sayegh HE Benslimane L Nouini Y. Sarcomatoid carcinoma of the urinary bladder: analysis of five cases and literature review. Pan Afr Med J. (2020) 36:369. 10.11604/pamj.2020.36.369.25036 33235646 PMC7666685

[5] Karmakar S Ansari MHG Thakur S Rai DK. Sarcomatoid carcinoma of the lung. Lung India. (2021) 38(3):266–8. 10.4103/lungindia.lungindia_67_20 33942753 PMC8194439

[6] Liao SH Su TH Jeng YM Liang PC Chen DS Chen CH Clinical manifestations and outcomes of patients with sarcomatoid hepatocellular carcinoma. Hepatol. (2019) 69(1):209–21. 10.1002/hep.30162 30014620

[7] Okabayashi T Shima Y Iwata J Iiyama T Sumiyoshi T Kozuki A Surgical outcomes for 131 cases of carcinosarcoma of the hepatobiliary tract. J Gastroenterol. (2014) 49(6):982–91. 10.1007/s00535-013-0882-2 24162331

[8] Kan A Guo RP. The prognosis of subsequent surgical treatment in patients with sarcomatoid carcinoma in the liver: a retrospective study. Int J Surg. (2018) 55:145–51. 10.1016/j.ijsu.2018.05.736 29860126

[9] Koda M Maeda Y Matsunaga Y Mimura K Murawaki Y Horie Y. Hepatocellular carcinoma with sarcomatous change arising after radiofrequency ablation for well-differentiated hepatocellular carcinoma. Hepatol Res. (2003) 27(2):163–7. 10.1016/s1386-6346(03)00207-9 14563432

[10] Komada N Yamagata M Komura K Hayashi K Maruyama T Kataoka H Hepatocellular carcinoma with sarcomatous change arising in primary biliary cirrhosis. J Gastroenterol. (1997) 32(1):95–101. 10.1007/bf01213303 9058302

[11] Kojiro M Sugihara S Kakizoe S Nakashima O Kiyomatsu K. Hepatocellular carcinoma with sarcomatous change: a special reference to the relationship with anticancer therapy. Cancer Chemother Pharmacol. (1989) 23(1):S4–8. 10.1007/bf00647229 2466583

[12] Lu J Zhang J Xiong XZ Li FY Ye H Cheng Y Primary hepatic sarcomatoid carcinoma: clinical features and prognosis of 28 resected cases. J Cancer Res Clin Oncol. (2014) 140(6):1027–35. 10.1007/s00432-014-1641-3 24647927 PMC11823726

[13] Zhang H Chai S Chen L Wang Y Cheng Y Fang Q Mri features of hepatic sarcomatoid carcinoma different from hepatocellular carcinoma and intrahepatic cholangiocarcinoma. Front Oncol. (2021) 11:611738. 10.3389/fonc.2021.611738 34221954 PMC8247642

[14] Wang QB Cui BK Weng JM Wu QL Qiu JL Lin XJ. Clinicopathological characteristics and outcome of primary sarcomatoid carcinoma and carcinosarcoma of the liver. J Gastrointest Surg. (2012) 16(9):1715–26. 10.1007/s11605-012-1946-y 22767081

[15] Tang Y Zhang T Zhao Y Chen Z Ma X. Development and validation of a comprehensive radiomics nomogram for prognostic prediction of primary hepatic sarcomatoid carcinoma after surgical resection. Int J Med Sci. (2021) 18(7):1711–20. 10.7150/ijms.53602 33746587 PMC7976557

[16] Ma Q Jiang L Bonda S Luo D Zhang W. A rare case of hepatic sarcomatoid carcinoma: exceeding expectations in a stage iv primary hepatic sarcomatoid carcinoma patient. Int J Clin Exp Pathol. (2019) 12(1):378–83. 31933755 PMC6943997

[17] Gu KW Kim YK Min JH Ha SY Jeong WK. Imaging features of hepatic sarcomatous carcinoma on computed tomography and gadoxetic acid-enhanced magnetic resonance imaging. Abdom Radiol (NY). (2017) 42(5):1424–33. 10.1007/s00261-016-1038-7 28078380

[18] Shi D Ma L Zhao D Chang J Shao C Qi S Imaging and clinical features of primary hepatic sarcomatous carcinoma. Cancer Imaging. (2018) 18(1):36. 10.1186/s40644-018-0171-7 30314525 PMC6186076

[19] Seo N Kim MJ Rhee H. Hepatic sarcomatoid carcinoma: magnetic resonance imaging evaluation by using the liver imaging reporting and data system. Eur Radiol. (2019) 29(7):3761–71. 10.1007/s00330-019-06052-8 30859282

[20] Koo HR Park MS Kim MJ Lim JS Yu JS Jin H Radiological and clinical features of sarcomatoid hepatocellular carcinoma in 11 cases. J Comput Assist Tomogr. (2008) 32(5):745–9. 10.1097/RCT.0b013e3181591ccd 18830104

[21] Honda H Hayashi T Yoshida K Takenaka K Kaneko K Fukuya T Hepatocellular carcinoma with sarcomatous change: characteristic findings of two-phased incremental ct. Abdom Imaging. (1996) 21(1):37–40. 10.1007/s002619900006 8672970

[22] Kim SA Lee JM Lee KB Kim SH Yoon SH Han JK Intrahepatic mass-forming cholangiocarcinomas: enhancement patterns at multiphasic ct, with special emphasis on arterial enhancement pattern–correlation with clinicopathologic findings. Radiol. (2011) 260(1):148–57. 10.1148/radiol.11101777 21474703

[23] Rossi G Cavazza A Sturm N Migaldi M Facciolongo N Longo L Pulmonary carcinomas with pleomorphic, sarcomatoid, or sarcomatous elements: a clinicopathologic and immunohistochemical study of 75 cases. Am J Surg Pathol. (2003) 27(3):311–24. 10.1097/00000478-200303000-00004 12604887

[24] Shen XZ Liu F. Primary sarcomatoid carcinoma of the mandibular gingiva: clinicopathological and radiological findings. Singapore Med J. (2014) 55(9):e152–5. 10.11622/smedj.2014131 25273946 PMC4293949

[25] Giordano G Berretta R Silini E. Primary pure spindle cell carcinoma (sarcomatoid carcinoma) of the ovary: a case report with immunohistochemical study. Diagn Pathol. (2016) 11(1):70. 10.1186/s13000-016-0521-3 27491291 PMC4974787

[26] Leng Q Xiang XI Tang Y Yang Y Qiu LI. Primary hepatic sarcomatoid carcinoma: a case report. Exp Ther Med. (2015) 10(3):1145–8. 10.3892/etm.2015.2599 26622454 PMC4533145

[27] Shi Y Rojas Y Zhang W Beierle EA Doski JJ Goldfarb M Characteristics and outcomes in children with undifferentiated embryonal sarcoma of the liver: a report from the national cancer database. Pediatr Blood Cancer. (2017) 64(4). 10.1002/pbc.26272 PMC5333454 27781381

